# C-Terminal-Pro-Endothelin-1 Adds Incremental Prognostic Value for Risk Stratification After Ischemic Stroke

**DOI:** 10.3389/fneur.2020.629151

**Published:** 2021-01-27

**Authors:** Laura P. Westphal, Juliane Schweizer, Felix Fluri, Gian Marco De Marchis, Mirjam Christ-Crain, Andreas R. Luft, Mira Katan

**Affiliations:** ^1^Department of Neurology, University Hospital Zurich and University of Zurich, Zurich, Switzerland; ^2^Department of Neurology, Stiftung Rehabilitation Heidelberg (SRH) Health Center Bad Wimpfen, Bad Wimpfen, Germany; ^3^Department of Neurology, University Hospital Basel and University of Basel, Basel, Switzerland; ^4^Department of Endocrinology, Diabetes and Metabolism, University Hospital Basel and University of Basel, Basel, Switzerland

**Keywords:** stroke, biomarker, C-terminal-pro-endothelin-1, outcome, mortality, risk stratification

## Abstract

**Background and Aims:** Endothelins have shown to play a role in the pathophysiology of ischemic stroke. We aimed at evaluating the incremental prognostic value of C-terminal-pro-endothelin-1 (CT-pro-ET-1) in a well-described cohort of acute stroke patients.

**Methods:** We performed serial measurements of CT-pro-ET-1 in 361 consecutively enrolled ischemic stroke patients and assessed functional outcome and mortality after 90 days. As we found peak levels of CT-pro-ET-1 and the most prominent association with mortality on day 1 after admission (*n* = 312), we focused on this time point for further outcome analyses. We calculated logistic regression and cox proportional hazards models to estimate the association of CT-pro-ET-1 with our outcome measures after adjusting for demographic and clinical risk factors. To evaluate the incremental value of CT-pro-ET-1, we calculated the area under the receiver operating characteristics (AUC) curve and the continuous net reclassification index (cNRI) comparing the model with and without the biomarker CT-pro-ET-1.

**Results:** In the univariate analysis CT-pro-ET-1 with a peak on day 1 after admission was associated with unfavorable outcome with an OR of 1.32 (95% CI, 1.16–1.51, *p* < 0.001) and with mortality with a HR of 1.45 (95% CI, 1.29–1.63, *p* < 0.001). After adjusting, CT-pro-ET-1 remained an independent predictor of mortality with an adjusted HR of 1.50 (95% CI, 1.29–1.74, *p* < 0.001), but not for functional outcome. Adding CT-pro-ET-1 to the cox-regression model for mortality, the discriminatory accuracy improved from 0.89 (95% CI, 0.84–0.94) to 0.92 (95% CI, 0.88–0.96) *p* < 0.001, and the cNRI was 0.72 (95% CI, 0.17–1.13).

**Conclusion:** CT-pro-ET-1 with a peak level on day 1 was an independent predictor of mortality adding incremental prognostic value beyond traditional risk factors.

## Introduction

Blood based biomarkers in the setting of acute ischemic stroke are in demand to optimize risk stratification and treatment decisions of stroke patients. Endothelins are a group of vasoactive endogenous peptides existing in three isoforms CT-pro-ET-1, −2, and −3 (1). CT-pro-ET-1 is expressed in endothelial cells, vascular smooth muscles and the central nervous system mediating vaso- and bronchoconstriction with a long duration of action (1). CT-pro-ET-1-synthesis can be stimulated via humoral factors, such as cytokines and activated platelets, homeostatic factors, such as hypoxia and hypovolemia, and also mechanically via tangential sheering-stress of arteries (1–3). Normal plasma levels range between 0.4 and 8.1 pg/ml (4). Elevated CT-pro-ET-1 levels have been shown to be linked to unfavorable outcome in ischemic stroke in a recent pilot study of 60 patients (5).

We aimed to evaluate the incremental prognostic value of CT-pro-ET-1 in a well-described, larger and independent validation cohort of acute stroke patients compared to the above-mentioned studies. Due to the vasoconstrictive effect of CT-pro-ET-1, we hypothesized an association of CT-pro-ET-1 with unfavorable outcome and mortality in acute ischemic stroke.

## Materials and Methods

### Study Design and Setting

This report adheres to the consolidated standards for the reporting of observational studies. In this prospective cohort-study we enrolled 361 ischemic stroke patients (6) and measured CT-pro-ET-1 on admission, day 1, 3, and 5 (see below). Patients were included with a clinically diagnosed acute ischemic stroke referring to the World Health Organization criteria (7) and a symptom onset within 72 h. The local Ethics Committee approved the study protocol. Informed consent was obtained in all patients. The study was conducted according to the World Medical Association Declaration of Helsinki. The primary outcome of the study was defined as favorable functional outcome after 90 days assessed by the modified Rankin Scale (mRS) score (8), (0–2 favorable, 3–6 unfavorable outcome), whereas secondary outcome was defined as death from any cause within a 90-day-follow-up period.

### Clinical Variables and Imaging

On admission to hospital, demographic and clinical data, laboratory findings, comorbidities assessed by the Charlson comorbidity index (CCI) and known cardiovascular risk factors were registered (Table 1). The severity of stroke was assessed by the National Institutes of Health Stroke Scale (NIHSS) score (9) performed by a trained stroke physician (0–42 points with higher scores indicating an increased stroke severity). We assessed the clinical stroke syndrome by applying the Oxford Community Stroke Project (10). Stroke causative factors were included after a cardiovascular work-up according to the Trial of Org 10172 in Acute Stroke Treatment (TOAST) classification (11). The assessment of outcome parameters was performed by two trained medical students blinded to CT-pro-ET-1 levels with follow-up interviews with the patient or, if not possible, with the closest relative or family physician. Magnetic resonance diffusion-weighted imaging (MR-DWI) lesion volumes were measured by consensus of two experienced raters unaware of clinical and laboratory findings. A semi-quantitative method validated for ischemic stroke lesions was used to calculate the lesion size (12). Lesions were categorized into three sizes to represent typical stroke patterns: (1) small lesion with a volume of <10 mm^3^, (2) medium lesion of 10–100 mm^3^, (3) large lesion with a volume of more than 100 mm^3^ (13).

**Table 1:** Baseline characteristics of stroke patients, stratified by mortality after 90 days.

		**Mortality**		
	**All**	**Alive**	**Dead**	***p***
	**(*n* = 362)**	**(*n* = 317)**	**(*n* = 44)**	
**Demographic factors**				
Age, median (IQR)	75 (63–83)	74 (61–82)	83 (78–87)	**<0.001**
Female sex, *n* (%)	149 (41)	128 (40)	21 (48)	n.s.
**Risk factors**, ***n*** **(%)**				
Hypertension	276 (76)	238 (75)	37 (84)	n.s.
Atrial fibrillation	75 (21)	57 (18)	18 (41)	**0.001**
Current smoking	125 (35)	113 (36)	11 (25)	n.s.
Diabetes mellitus	71 (20)	62 (20)	9 (20)	n.s.
Coronary heart disease	91 (25)	74 (23)	17 (39)	**<0.05**
Dyslipidemia	93 (26)	82 (26)	35 (25)	n.s.
Prior cerebrovascular event	88 (24)	80 (25)	8 (18)	n.s.
Modified Charlson Index (IQR)	1 (0–2)	1 (0–2)	1 (0–2)	n.s.
**Clinical data, median (IQR)**				
NIHSS on admission	5 (2–10)	4 (2–8)	17 (8–25)	**<0.001**
**Laboratory values, median (IQR)**				
CT-pro-ET-1 day 0 in pg/ml	8.4 (7.3–9.6)	8.4 (7.3–9.4)	9.6 (8.3–10.9)	**<0.001**
CT-pro-ET-1 day 1 (^*^, *n* = 312)	8.5 (7.7–9.6)	8.4 (7.6–9.4)	10.2 (9.2–11.4)	**<0.001**
CT-pro-ET-1 day 3 (^*^)	8.3 (7.6–9.3)	8.3 (7.6–9.2)	9.8 (8.4–11.4)	**<0.001**
CT-pro-ET-1 day 5 (^*^)	8.2 (7.4–9.4)	8.1 (7.4–9.2)	10.1 (7.8–14.2)	**0.001**
**Lesion size on MRI, DWI (*n* = 198), *n* (%)**				
Small (1–10 mm^3^) – size 1	136 (69)	131 (70)	4 (36)	**<0.05**
Medium (>10–100 mm^3^) – size 2	50 (25)	46 (25)	4 (36)	n.s.
Large (>100 mm^3^) – size 3	12 (6)	9 (5)	3 (27)	**<0.05**
**Stroke etiology**, ***n*** **(%)**				
Large-vessel disease	65 (18)	60 (19)	5 (11)	n.s.
Cardio-embolic (^**^)	131 (36)	111 (35)	20 (46)	n.s.
Small-artery disease	55 (15)	54 (17)	1 (2)	**<0.01**
Multiple causes	17 (5)	15 (5)	1 (2)	n.s.
Undetermined	94 (26)	77 (24)	17 (39)	**<0.05**

* *Data normalization by square root model*;

** *including atrial fibrillation, atrial flutter, congestive heart failure, patent foramen ovale; IQR, Interquartile range; NIHSS, National Institutes of Health Stroke Scale; MR, Magnetic Resonance; DWI, Diffusion Weighted Imaging; p, p-value; n.s., not significant. The bold values highlight significant values*.

### Assays

Blood samples of the acute stroke patients were taken on admission (i.e., day 0) within 0–72 h from symptom onset with a vast majority of samples (74%, *n* = 267) taken until 12 h after symptom onset. Additionally to admission (*n* = 335), samples of CT-pro-ET-1 were taken on day 1 (*n* = 312), day 3 (*n* = 264) and day 5 (*n* = 262). The routine blood analyses were recorded and plasma stored at −80° Celsius. For the analyses a single batch with a commercial sandwich immunoluminometric assay (B.R.A.H.M.S LUMItest CT-proAVP, B.R.A.H.M.S AG, Henningsdorf/Berlin, Germany) was applied as described in detail elsewhere (12). The assay (mean reference range, 44.3 ± 10.6 pmol/l) has an analytical detection limit of 0.4 pmol/l (14).

### Statistical Analysis

Discrete variables are summarized as counts (percentages), continuous variables as medians and interquartile ranges (IQR). To obtain normal distribution for skewed variables (i.e., CT-pro-ET-1 concentrations), we transformed the data by taking the square root. The Fisher‘s-exact test, respectively the Mann-Whitney U test were applied for two-group and the Kruskal Wallis test for multiple group comparisons. For the analysis of CT-pro-ET-1 and its association with stroke severity defined by the NIHSS on admission, we dichotomized patients into NIHSS ≤6 vs. NIHSS ≥7 in line with previous publications (6, 13). For the analysis of the association of CT-pro-ET-1 with lesion size, we conducted a bivariate regression analysis in the subgroup of patients with available information on lesion size.

We calculated logistic regression and cox proportional hazards models adjusting for significant outcome predictors. In the multivariate model analyzing mortality at 90 days after stroke onset, we adjusted for all risk factors, which where significant in the univariate analysis after bonferroni correction for multiple testing. These variables were chosen according to their magnitude of association with mortality in the univariate analysis and based on the Bonferroni corrected significance level of *p* < 0.00217.

To assess the discriminatory accuracy and incremental value of CT-pro-ET-1 beyond known risk factors, Receiver Operating Characteristic (ROC) curves and the area under the ROC curve (AUC) as an overall discriminatory measure were calculated for the model with and without CT-pro-ET-1 on day 1. The likelihood ratio test was used to compare the AUCs of nested vs. whole models. The whole model included all predictors that remained significant in the multivariate model. In addition, the cNRI was assessed considering only those changes in estimated prediction probabilities that imply a change from one risk category to another. A cut-off was identified by classifying sensitivity and specificity for CT-pro-ET-1 levels choosing the cut-off at the highest possible sensitivity for detection with still enough specificity to predict mortality. For Kaplan-Meier survival estimates, patients were stratified by the selected cut-off level for CT-pro-ET-1 of <8.8 pg/ml (57%) and ≥8.8 pg/ml (43%) measured on day 1 after admission. Groups were compared by means of the log-rank test. *P*-values ≤0.05 were considered to be statistically significant. All calculations were performed using STATA 14.1.

## Results

An acute ischemic stroke was diagnosed in 362 patients with 361 completing follow-up. These 361 patients were analyzed for baseline characteristics and stratified for mortality after 90 days as shown in Table 1.

### CT-Pro-ET-1 Over Time

**Figure 1:**
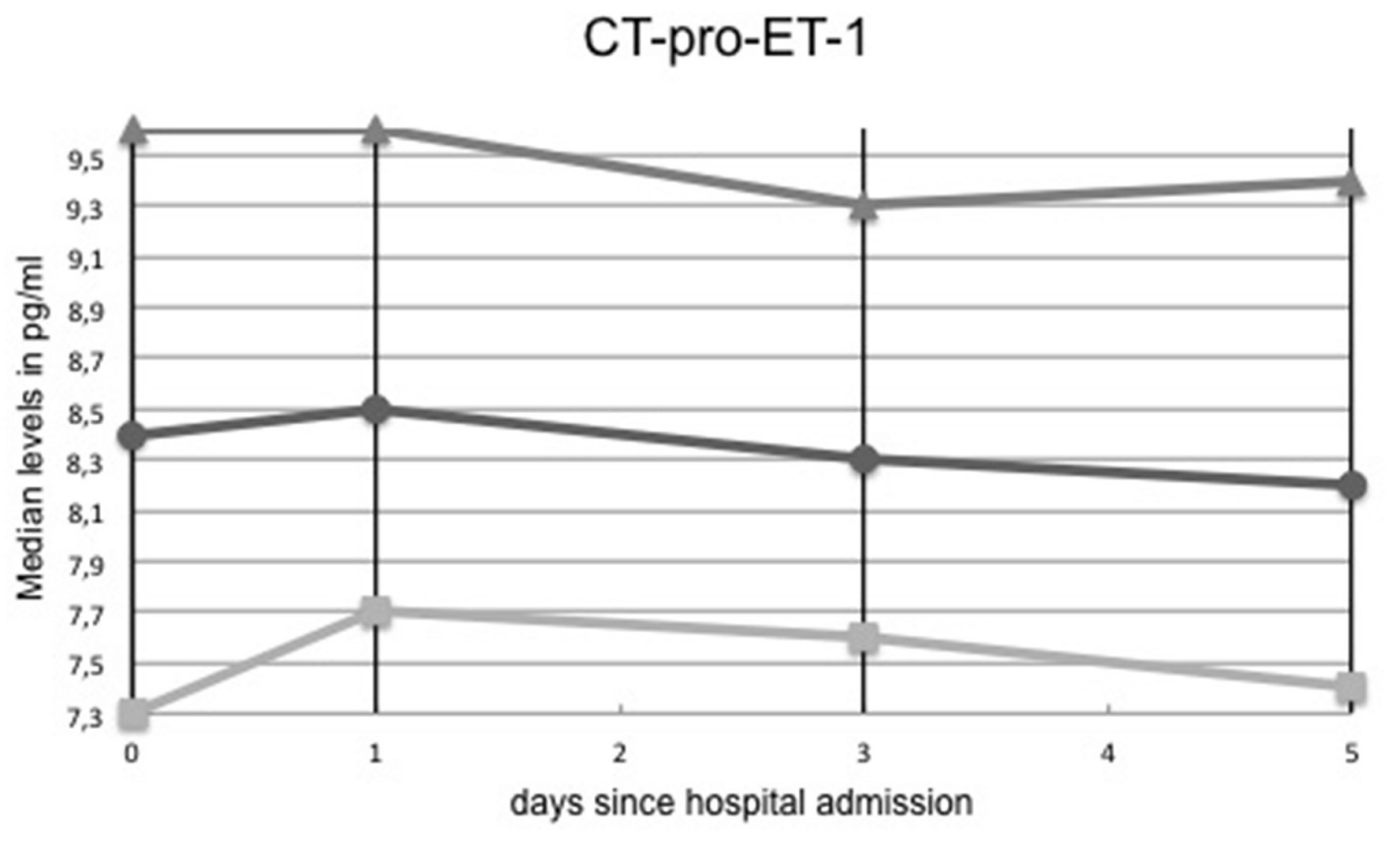
CT-pro-ET-1 levels over time. Circles indicate median CT-pro-ET-1 levels, squares lower 95%-confidence interval limits and arrows upper confidence interval limits, respectively.

When assessing CT-pro-ET-1 sequentially on admission (i.e., day 0), day 1, 3, and 5, we observed a nominal increase of CT-pro-ET-1 over time with a peak on day 1 (8.5 pg/ml, IQR 7.7–9.6) followed by a gradual decrease over the next days with a minimum on day 5 (8.2 pg/ml, IQR 7.4–9.4), see Table 1 and Figure 1. Thus, for further analyses, we concentrated on the predictive value of CT-pro-ET-1 on day 1 as at this point of time after stroke the association of CT-pro-ET-1 levels with mortality within 90 days was most prominent.

CT-pro-ET-1 levels on day 1 were available in 312 out of 362 patients. When conducting a sensitivity analysis between the original cohort of 361 patients, which was used for the analysis of baseline characteristics, and the cohort with available CT-pro-ET-1 measurements on day 1 (*n* = 312), we found no significant difference between both cohorts regarding the distribution of baseline risk factors (data not shown).

### CT-Pro-ET-1 and Its Association With Baseline Risk Factors for Mortality

CT-pro-ET-1 levels on day 1 were higher in patients suffering from severe strokes compared to moderate strokes, but this difference was not statistically significant (NIHSS ≤6: CT-pro-ET-1 on day 1 8.4 pg/ml, IQR 7.7–9.4, vs. NIHSS ≥7: 8.8 pg/ml, IQR 7.6–9.9, *p* = 0.16).

A moderate to large ischemic lesion (>10 mm^3^ on MR-DWI) was associated with higher CT-pro-ET-1 levels compared to small lesions (<10 mm^3^: CT-pro-ET-1 on day 1 8.1 pg/ml, IQR 7.5–8.8, vs. >10 mm^3^: 8.7 pg/ml, IQR 7.6–10.2, *p* = 0.02) in a subgroup of patients with available imaging (*n* = 182).

### CT-Pro-ET-1 and Its Association With Stroke Etiology

We found only a borderline association of CT-pro-ET-1 levels on day 1 and cardio-embolic stroke etiology according to the TOAST criteria. However, after adjustment for multiple testing (comparison of all TOAST subgroups with each other), the association of CT-pro-ET-1 with cardio-embolic stroke etiology could not be confirmed (*p* > 0.05).

### Prediction of Functional Outcome and Mortality After 90 Days

CT-pro-ET-1 on day 1 was associated with an unfavorable functional outcome with an odds ratio (OR) of 1.32 (95% CI, 1.16–1.51). Adjustment for significant outcome predictors (age, NHISS on admission and atrial fibrillation) in the multivariate analysis attenuated the association with an OR of 1.05 (95% CI, 0.88–1.25, *p* = 0.59).

**Figure 2:**
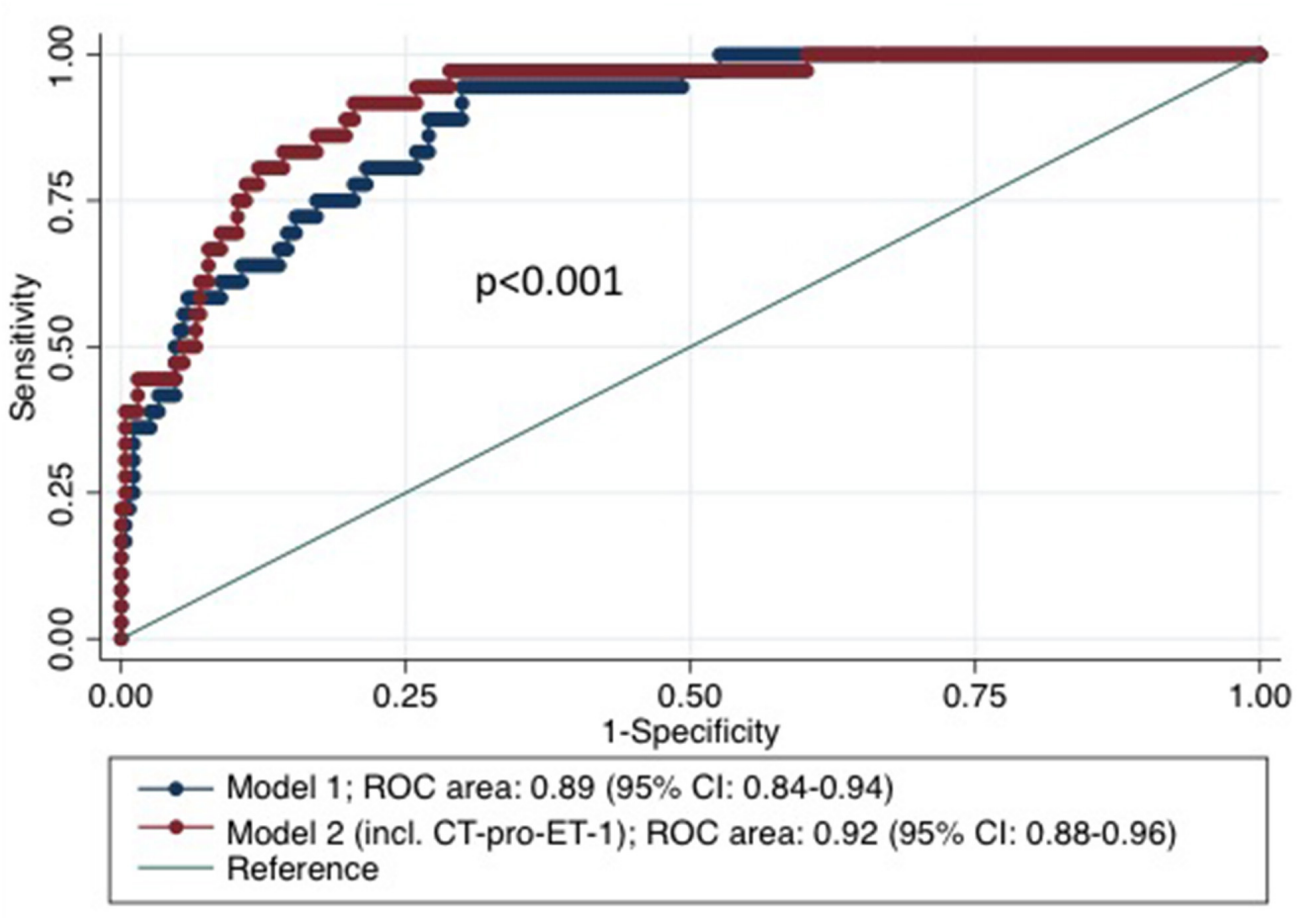
Receiver Operator Characteristic (ROC)-curves based on regression models for mortality within 3 months after stroke with and without CT-pro-ET-1. Model 1 represents the cox proportional hazards model for mortality within 3 months after stroke including the risk factors age and NIHSS on admission. Model 2 represents the analysis adding CT-pro-ET-1 on day 1 after admission to the model.

**Figure 3:**
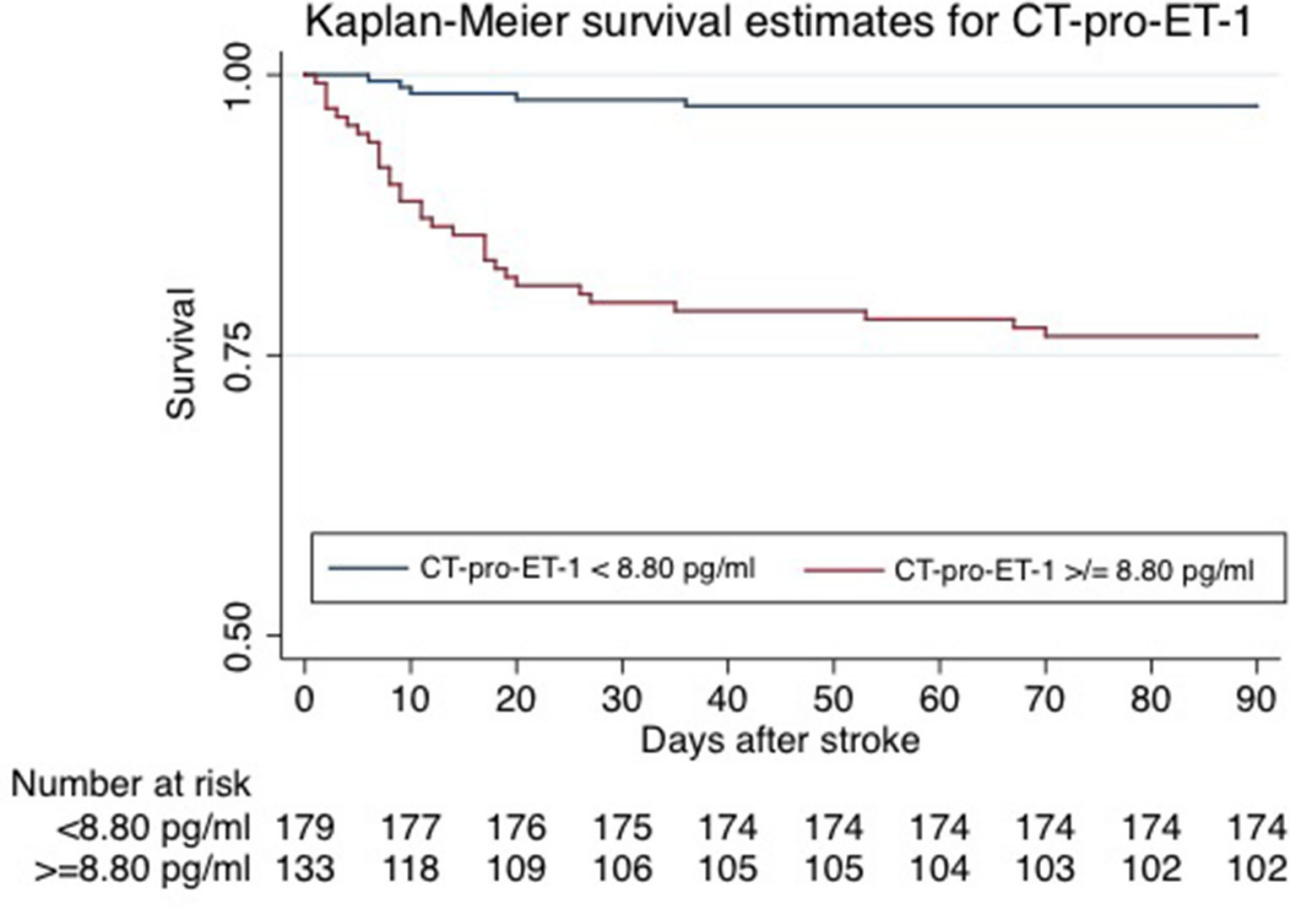
Kaplan-Meier survival estimates of patients stratified by CT-pro-ET-1 on day 1 after admission. Kaplan-Meier survival curves of patients stratified per CT-pro-ET-1 cut-off levels <8.80 and ≥8.80 pg/ml differed significantly (*p* = <0.001, log-rank test).

CT-pro-ET-1 on day 1 was associated with mortality with a hazards ratio (HR) of 1.45 (95% CI, 1.29–1.63). After adjusting, CT-pro-ET-1 remained an independent predictor of mortality with an adjusted HR of 1.50 (95% CI, 1.29–1.74, *p* < 0.001, Table 2). CT-pro-ET-1 on day 1 ≥8.8 pg/ml had a sensitivity of 89% and a specificity of 63% to predict mortality. Adding CT-pro-ET-1 to the regression model for mortality, the discriminatory accuracy improved from 0.89 (95% CI, 0.84–0.94) to 0.92 (95% CI, 0.88–0.96), *p* < 0.001, see Figure 2. The combination of CT-pro-ET-1 with the regression model led to a cNRI of 0.72 (95% CI, 0.17–1.13). Overall, Kaplan-Meier survival curves of patients stratified according to the above-mentioned CT-pro-ET-1 cut-off level <8.8 and ≥8.8 pg/ml differed (*p* < 0.001, log-rank test) (Figure 3).

**Table 2:** Multivariate analysis: cox proportional hazards model for survival after 90 days.

**Predictors**	**Mortality**		
	**HR**	**95% CI**	***P***
CT-pro-ET-1 day 1 (^*^)	1.5	1.29–1.74	**<0.001**
Age (increase per unit)	1.04	1.01–1.08	**0.006**
NIHSS (increase per unit)	1.12	1.09–1.16	**<0.001**
Atrial fibrillation	0.54	0.24–1.21	0.13

* *Data normalization by square root model; HR, hazard ratio; CI, confidence interval; p, p-value; NIHSS, National Institutes of Health Stroke Scale. The bold values highlight significant values*.

In a bivariate analysis within the subgroup of patients with available MRI data (*n* = 182) CT-pro-ET-1 on day 1 also remained associated with mortality with an HR of 1.49 (95% CI 1.21–1.84, *p* < 0.001) after adjusting for lesion size.

## Discussion

This prospective single-center cohort study revealed the following key findings: CT-pro-ET-1 with peak levels on day 1 after admission was positively associated with unfavorable outcome and most remarkably and independently with mortality after 3 months. CT-pro-ET-1 improved the discriminatory accuracy of the NIHSS alone as well as compared to the multivariate model without the biomarker as shown by an increase of the respective AUC and cNRI.

Circulating CT-pro-ET-1 levels have been described to be very low in normal conditions (15). During acute ischemic events several mechanisms have been discussed leading to an increase of CT-pro-ET-1. Among these, CT-pro-ET-1 is released as an acute phase reactant following severe physical stress caused by hypoxia (16). Furthermore, the hypercoagulability and platelet activation in ischemic lesions have been described to accelerate pre-proendothelin production (17). Via activation of inflammatory cells such as neutrophils, mast cells and macrophages CT-pro-ET-1 might exert pro-inflammatory effects (18) and thus besides the potent vasoconstrictive capability this pro-inflammatory aspect may contribute also to a worse outcome in stroke patients.

CT-pro-ET-1 has been linked particularly to cardiovascular disease previously. In the Leicester Acute Myocardial Infarction Peptide (LAMP) Study Khan et al. found increased CT-pro-ET-1 levels to be independently associated with a higher rate of heart failure and mortality in a large cohort of 983 acute myocardial infarction patients (19). In line with our results, peak plasma concentrations have been described to appear with a little delay on day 2 after myocardial infarction (19). Adverse outcome effects in cardiovascular disease have been linked to a reduction of coronary blood flow (20).

However, regarding the association of CT-pro-ET-1 and unfavorable outcome in acute ischemic stroke, the available studies remain to some extent controversial. A previous smaller pilot study consisting of 60 stroke patients has shown higher CT-pro-ET-1 levels associated with unfavorable outcome and mortality (5), whereas an older case-control study did not show a significant difference in CT-pro-ET-1 levels between healthy controls and ischemic stroke patients (21). Another case-control study with 30 sex- and age-matched patients found in line with our results higher CT-pro-ET-1 plasma levels on admission compared to day 7 after stroke onset and healthy controls, but could not find any correlation with infarct size, stroke severity or degree of clinical neurological deficit (22).

The strengths of this study are the prospective study design, the clinically well-characterized relatively large stroke patient cohort, a very low lost to follow-up-rate and blinded CT-pro-ET-1-measures. Additionally, we have shown serial measurements of CT-pro-ET-1 in the first hours up to 5 days after stroke onset in order to deduct the best time for measurement after stroke. By defining a cut-off value for CT-pro-ET-1 with levels > 8.8 pg/ml representing a 89%-sensitivity and 63%-specificity to predict mortality, the parameter could be applied for risk stratification in a clinical setting after external validation of this cut-off. This additional information beyond traditional risk factors could help physicians in clinical decision making, specifically to triage ischemic stroke patients, e.g., for intensified monitoring with regards to post-stroke complications. As we could also prove the association of CT-pro-ET-1 with mortality within 3 months after stroke onset in the subgroup of patients with available MRI data, the predictive value of CT-pro-ET-1 on mortality can be considered reliable supporting the independent additive prognostic information gained by measuring the biomarker. When compared to other thromboinflammatory biomarkers from the literature such as S100B, which has been associated with tissue damage, post-stroke infections and consecutively mortality (23), CT-pro-ET-1 predicted overall mortality in ischemic stroke patients independently of known risk factors.

As a limitation of the study, the role of CT-pro-ET-1 in hyper-acute treatment decisions is at least partly restricted since the peak of CT-pro-ET-1 was measured on day 1 after admission. Furthermore, time points of serial blood collections of CT-pro-ET-1 were classified in days after hospital admission and not in hours after stroke onset except for the day of hospital admission (day 0). However, as the majority of blood samples were taken until 12 h after stroke onset (74%), we can assume that for subsequent blood samples the days after hospital admission correspond largely with the days after stroke onset. Serial measurements of CT-pro-ET-1 levels in stroke survivors revealed only marginal changes over time (see Table 1), most likely as these patients had less severe strokes and therefore less complications over time. Thus, the stimulus of CT-pro-ET-1 production over time stayed stable in the group of stroke survivors. Additionally, since we are the first to propose a cut-off, there is need for an external validation of exactly the proposed cut off. Yet, as mentioned smaller studies in the past have shown overall association with mortality after stroke, therefore our study can also be interpreted as external validation of previous smaller studies.

In summary, CT-pro-ET-1 can be considered as an independent predictor of mortality in acute ischemic stroke patients. Further studies should assess the suggested cut-off value in larger and multi-center validation cohorts to prove the benefit in clinical decision making.

## Data Availability Statement

Anonymized data will be shared by request from any qualified investigator.

## Ethics Statement

The study involving human participants was reviewed and approved by the local Ethics Committee of Basel, Switzerland. The patients/participants provided their written informed consent to participate in this study.

## Disclosure

MK received the KITs for the measurement of CT-pro-ET-1 from BRAHMS GmbH, Henningsdorf, Germany.

## Author Contributions

LPW conducted data processing and interpretation, performed the statistical analysis, and wrote the first draft of the manuscript. JS was involved in the statistical analysis. FF, GMDM, and MC-C were involved in data collection and critically revised the manuscript. ARL critically revised the manuscript. MK designed the study, was involved in data analysis, and critically revised the manuscript. All authors contributed to the article and approved the submitted version.

## Conflict of Interest

The authors declare that the research was conducted in the absence of any commercial or financial relationships that could be construed as a potential conflict of interest.
